# Safety and ARIA Results of the Oral Anti‐Amyloid Agent Valiltramiprosate from the Phase 3 APOLLOE4 Trial in APOE4/4 Homozygotes with Early AD

**DOI:** 10.1002/alz70861_108685

**Published:** 2025-12-23

**Authors:** Patrick Kesslak, Susan Abushakra, David Watson, Earvin Liang, Jerome Barakos, Rosalind MacLaine, Anton P. Porsteinsson, Marwan N. Sabbagh, Emer MacSweeney, Sharon Cohen, Mercè Boada, Susan Flint, Aidan Power, John A. Hey, Martin Tolar

**Affiliations:** ^1^ Alzheon Inc., Framingham, MA USA; ^2^ Alzheon, Inc., Framingham, MA USA; ^3^ Alzheimer's Treatment and Research Center, Wellington, FL USA; ^4^ Clario, San Mateo, CA USA; ^5^ Alzheon, Framingham, MA USA; ^6^ University of Rochester School of Medicine and Dentistry, Rochester, NY USA; ^7^ Barrow Neurological Institute, Phoenix, AZ USA; ^8^ Re‐Cognition Health, London, Greater London UK; ^9^ Toronto Memory Program, toronto, ON Canada; ^10^ Research Center and Memory Clinic, ACE Alzheimer Center Barcelona, Universitat Internacional de Catalunya, Barcelona Spain

## Abstract

**Background:**

Valiltramiprosate/ALZ‐801, an oral inhibitor of amyloid oligomer formation, was evaluated in a Phase 3 trial in APOE4/4 homozygotes with Early AD, with main results presented at this meeting (Power et al, 2025). APOE4 is a risk factor for cerebral amlyloid angiopathy (CAA) and ARIA. Tramiprosate (active agent in ALZ‐801) had shown no ARIA‐E in prior studies, allowing the APOLLOE4 trial to include subjects with any number of microhemorrhages (MH) and several siderosis lesions at screening MRI.

**Method:**

This 78‐week placebo‐controlled trial enrolled 325 homozygotes who received placebo or 265 mg BID. Anti‐coagulants were exclusionary. In addition to adverse event (AE) assessments, MRIs were performed every 26 weeks and evaluated by Clario Inc. for occurrence of ARIA with edema and/or microhemorrhage/siderosis (ARIA‐E, ARIA‐H).

**Result:**

Safety population (*N* =325) was 51% females, 89% Caucasian, mean age 68 years, MMSE 25.6, with MCI vs. Mild AD (39% vs 61%); 298 had screening MRI (149/arm provided ≥1 post‐dose MRI). At baseline, 32% had >1 MH (placebo, 32%, active, 31%); 11% in placebo and 8% in active arm had siderosis (maximum 4 and 5 lesions, respectively); and 5% in each arm had ≥ 10 MH. New ARIA‐E occurred during the study in 5 subjects in each arm (3%) and new macrohemorrhages in 2 subjects/arm. Increase in MH from low to high category (0, 1‐4, 5‐9, ≥10 MH) was seen in 14% placebo and 9% active arm. Radiographic ARIA were all mild except 1 severe (placebo) and 1 moderate (active); all were without symptoms. AEs with incidence >5% and >2x placebo rate were nausea (26%), weight decrease (14%), decreased appetite and vomiting (10% each); with lower incidence of headache, falls and dizziness in active arm.

**Conclusion:**

APOLLOE4 enrolled homozygotes with a high CAA burden who would be ineligibile for amyloid antibody treatments. Valiltramiprosate overall safety was favorable and consistent with prior studies. ARIA‐E incidence was similar to placebo, and ARIA‐H was lower than placebo with no symptomatic ARIA events over 78 weeks of treatment. Coupled with promising efficacy in MCI subjects, valiltramiprosate can provide a favorable benefit‐risk profile for the high‐risk APOE4/4 patients with unmet treatment need.

